# TSG-6 Secreted by Human Adipose Tissue-derived Mesenchymal Stem Cells Ameliorates DSS-induced colitis by Inducing M2 Macrophage Polarization in Mice

**DOI:** 10.1038/s41598-017-04766-7

**Published:** 2017-07-12

**Authors:** Woo-Jin Song, Qiang Li, Min-Ok Ryu, Jin-Ok Ahn, Dong Ha Bhang, Yun Chan Jung, Hwa-Young Youn

**Affiliations:** 1 Department of Veterinary Internal Medicine, College of Veterinary Medicine, Seoul National University, Seoul, 08826 Republic of Korea; 2 Department of Molecular and Cellular Biology, Samsung Biomedical Research Institute, Sungkyunkwan University School of Medicine, Suwon, Gyeonggi, 16419 Republic of Korea; 3 KPC Corporation, Gwangju, Gyeonggi, 12773 Republic of Korea

## Abstract

Previous studies have revealed that mesenchymal stem cells (MSCs) alleviate inflammatory bowel disease (IBD) by modulating inflammatory cytokines in the inflamed intestine. However, the mechanisms underlying these effects are not completely understood. We sought to investigate the therapeutic effects of human adipose tissue-derived (hAT)-MSCs in an IBD mouse model and to explore the mechanisms of the regulation of inflammation. Dextran sulfate sodium-induced colitis mice were infused with hAT-MSCs intraperitoneally and colon tissues were collected on day 10. hAT-MSCs were shown to induce the expression of M2 macrophage markers and to regulate the expression of pro- and anti-inflammatory cytokines in the colon. Quantitative real time-PCR analyses demonstrated that less than 20 hAT-MSCs, 0.001% of all intraperitoneally injected hAT-MSCs, were detected in the inflamed colon. To investigate the effects of hAT-MSC-secreted factors *in vitro*, transwell co-culture system was used, demonstrating that tumour necrosis factor-α-induced gene/protein 6 (TSG-6) released by hAT-MSCs induces M2 macrophages. *In vivo*, hAT-MSCs transfected with TSG-6 small interfering RNA, administered intraperitoneally, were not able to induce M2 macrophage phenotype switch in the inflamed colon and had no significant effects on IBD severity. In conclusion, hAT-MSC-produced TSG-6 can ameliorate IBD by inducing M2 macrophage switch in mice.

## Introduction

Inflammatory bowel disease (IBD) is an intractable autoimmune disease, leading to a chronic inflammation of the digestive system, which can be classified as either ulcerative colitis or Crohn’s disease, depending on the site and pattern of inflammation^1, 2^. Although the exact pathogenesis of IBD remains unknown, it is believed to be associated with genetic and environmental factors, as well as inflammatory responses towards gut flora^3, 4^. Intestinal inflammatory response is known to be regulated through the secretion of inflammatory cytokines, such as tumor necrosis factor (TNF)-α, interferon (IFN)-γ, transforming growth factor (TGF)-β, interleukin (IL)-1β, -4, -6, -10, -17 and -23 which are secreted by macrophages and T-cells^5^. Even though there are numerous patients worldwide who suffer from IBD, which leads to a diminished quality of life, effective treatment for IBD has not been developed yet.

Recent studies have demonstrated that mesenchymal stem cells (MSCs) show anti-inflammatory effects and can alleviate the symptoms in various inflammatory disease models, including rheumatoid arthritis, peritonitis, and pancreatitis, as well as IBD^6–9^. Currently, the mechanisms underlying the anti-inflammatory effects are being investigated, in order to establish the basis for effective clinical application of MSCs. Moreover, several studies reported that even when MSCs do not migrate directly to the site of inflammation or the injured tissue, they can still exert anti-inflammatory actions through secretory factors^10, 11^. TNF-α-induced gene/protein 6 (TSG-6) is one of the best-known secretory factors responsible for anti-inflammatory activity, and recently, it was demonstrated that it plays crucial roles in the regulation of inflammatory responses in IBD, peritonitis, myocardial infarction, lung injury, corneal injury, and skin wound healing^12–17^.

Macrophages represent one of the key immune cells of the innate immunity and play a role as the link with immune cells responsible for the acquired immune response, such as the lymphocytes^18^. According to several recently published studies, macrophages in the inflamed tissues can be classified into two distinct types: M1 and M2 macrophages^19, 20^. M1 macrophages trigger inflammatory response by secreting cytokines such as TNF-α and IL-1β, whereas M2 macrophages induce anti-inflammatory responses by secreting cytokines such as IL-10^21^. Recent *in vitro* and inflammation-induced animal model studies demonstrated that MSCs can induce M1 to M2 macrophage phenotypic switch^22–24^, but the mechanisms underlying this process are not clearly understood.

In this study, we assessed the effects of human adipose tissue-derived (hAT)-MSCs on a dextran sulfate sodium (DSS)-induced colitis model in mice, together with the effect on phenotypic switch in macrophages. Additionally, we aimed to elucidate the mechanisms underlying these processes.

## Results

### Intraperitoneally administered hAT-MSCs ameliorate IBD

**Figure 1:**
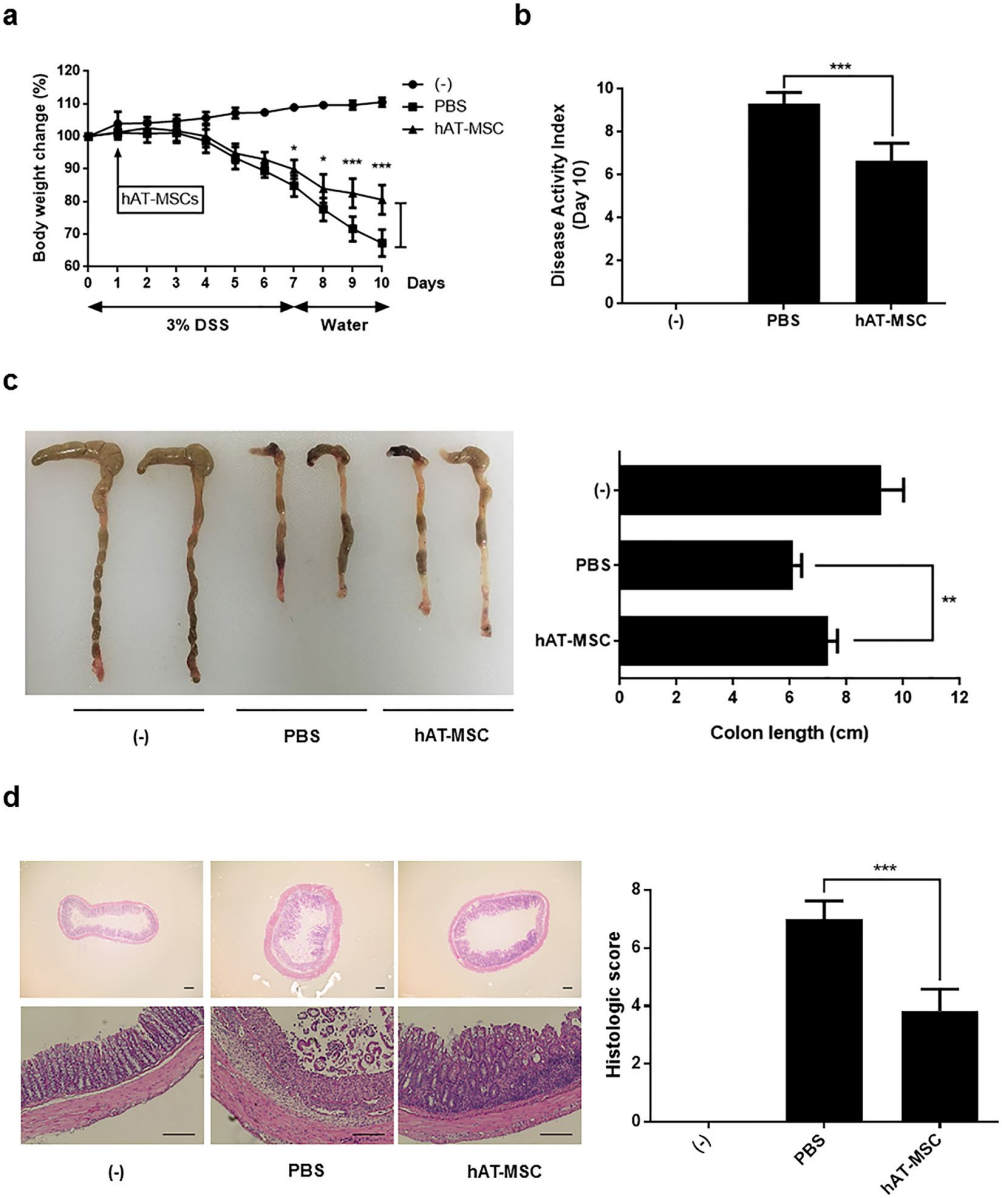
Intraperitoneally injected hAT-MSCs ameliorate IBD. DSS-induced colitis mice were infused hAT-MSCs or PBS (vehicle control) on day 1. (**a**) Body weight, measured every day and expressed as the relative change from day 0. (**b**–**d**) Mice were sacrificed on day 10 and (**b**) DAI, and (**c**) colon length, were assessed. (**d**) Representative H&E staining of the colon sections, and their histological scores are shown. Bars = 100 µm. Six to eight mice per group were used. Results are shown as mean ± standard deviation. *P < 0.05, **P < 0.01, ***P < 0.001.

Intraperitoneal injection of hAT-MSCs was shown to lead to a significant reduction in body weight loss, in comparison with that measured in mice injected with the phosphate-buffered saline (PBS) from day 7 (Fig. 1a). On day 10, the disease activity index (DAI) of mice treated with hAT-MSCs was significantly decreased, in comparison with that in the mice treated with PBS (Fig. 1b). To evaluate length and histology of the colon, mice were sacrificed on day 10. Compared with that in the PBS-treated group, colon length was significantly improved in hAT-MSC-treated group (Fig. 1c). Histological examination of the DSS-induced colitis mouse colons showed severe submucosal or transmural thickening, destruction of entire epithelium, and severe inflammatory cell infiltration. In contrast, the extent of bowel wall thickening, crypt damage, and the infiltration of inflammatory cells were reduced in colon sections obtained from mice injected with hAT-MSCs, compared with those in the PBS-treated mice (Fig. 1d). The analysis for haematoxylin and eosin (H&E)-stained colon sections showed a significant decrease in the histologic scores of hAT-MSC-treated group, in comparison with those in the PBS-treated group (Fig. 1d).

### hAT-MSCs reduce the inflammatory response by increasing the percentage of M2 macrophages in colon

**Figure 2:**
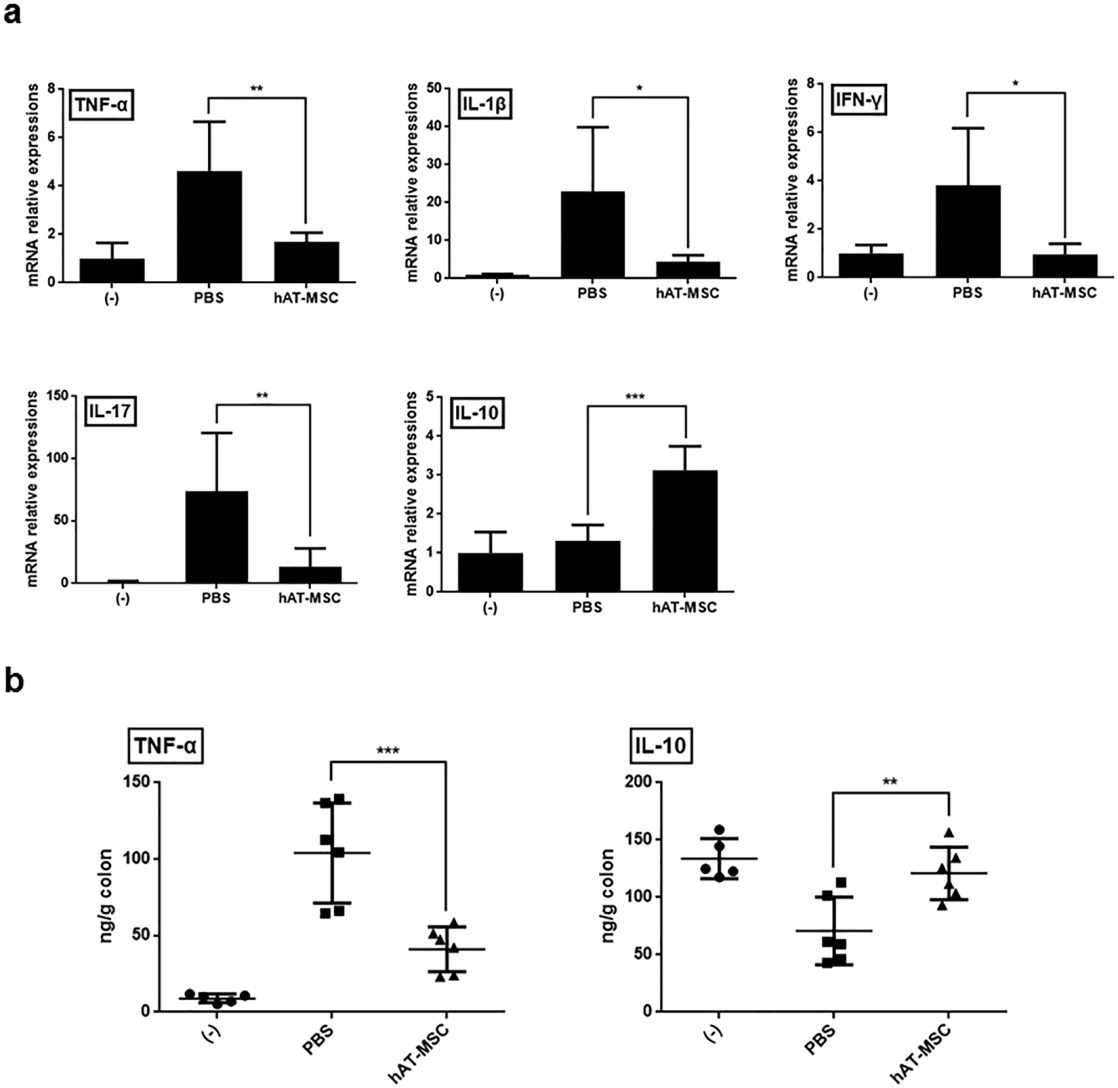
hAT-MSCs inhibit inflammatory response in the colon. (**a**) mRNA expression levels of pro- and anti-inflammatory cytokines in colons were determined by qRT-PCR. (**b**) Levels of TNF-α and IL-10 in colons were assessed using ELISA. Six to eight mice per group were used. Results are presented as mean ± standard deviation. *P < 0.05, **P < 0.01, ***P < 0.001.

We explored whether intraperitoneally injected hAT-MSCs could modulate inflammatory cytokines associated with the development of DSS-induced colitis. In colon tissues of PBS-treated mice with colitis, the mRNA expression levels of TNF-α, IL-1β, IFN-γ, and IL-17 were considerably increased, whereas that of IL-10 was only slightly increased (Fig. 2a). The administration of hAT-MSCs not only significantly decreased the expression level of TNF-α, IL-1β, IFN-γ, and IL-17, but also significantly increased the expression of IL-10 (Fig. 2a). Furthermore, the protein levels of TNF-α and IL-10 also exhibited a similar pattern in samples from the colitis mice (Fig. 2b).

**Figure 3:**
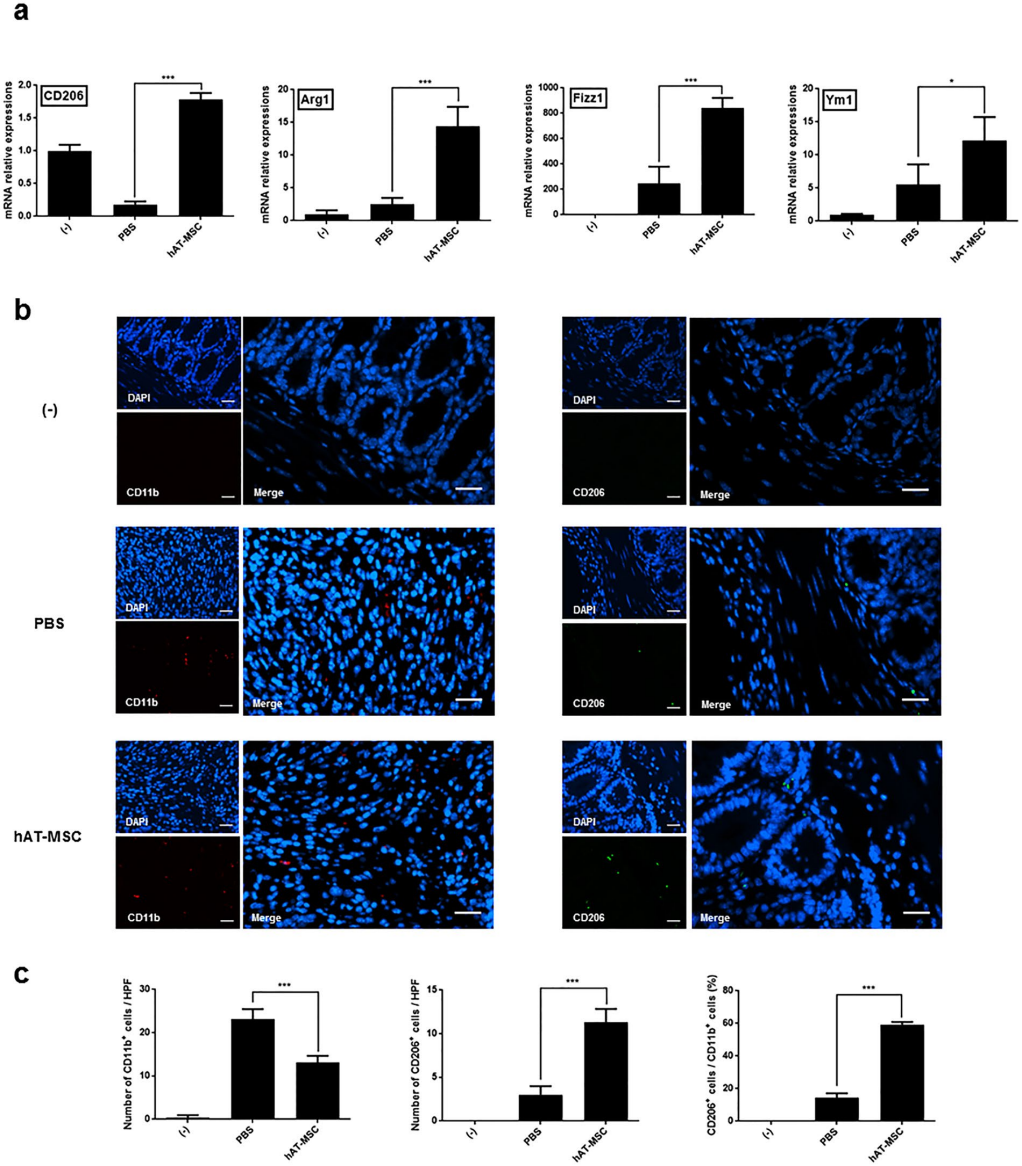
hAT-MSC administration leads to an increase in the percentage of M2 macrophages in the colon. (**a**) Expression levels of CD206, Arg1, Fizz1, and Ym1 were determined using qRT-PCR. (**b**) Representative immunofluorescence staining using anti-CD11b or anti-CD206 antibodies. Bars = 30 µm. (**c**) The number of CD11b- or CD206-positive cells within the inflammatory infiltrates, and the calculated percentage of CD206-positive cells among the CD11b-positive cells. Six to eight mice per group were used. Results are presented as mean ± standard deviation. *P < 0.05, ***P < 0.001.

Considering that hAT-MSCs may affect the activity of inflammatory cytokines, we examined the presence of M2 macrophages in the colon tissues. The expression levels of CD206, Arg1, Fizz1, and Ym1 (well-known M2 macrophage markers) were decreased or slightly increased in the colons of colitis model mice treated with PBS (Fig. 3a). However, the injection of hAT-MSCs led to a significant increase in the expression of M2 macrophage markers, compared with that in the PBS-treated mice (Fig. 3a). Furthermore, the total macrophages and M2 macrophages that infiltrated the colon tissue sections were detected using antibodies specific to CD11b and CD206, respectively (Fig. 3b). In addition, we calculated the percentage of CD206-positive cells among the CD11b-positive cells in colon sections from the same mice. The percentage of M2 macrophages in the PBS-treated group was 14.50% ± 2.65%, whereas it was 59.25% ± 1.71% in the hAT-MSC-treated group (Fig. 3c).

### Intraperitoneally injected hAT-MSCs do not migrate to the colon

**Figure 4:**
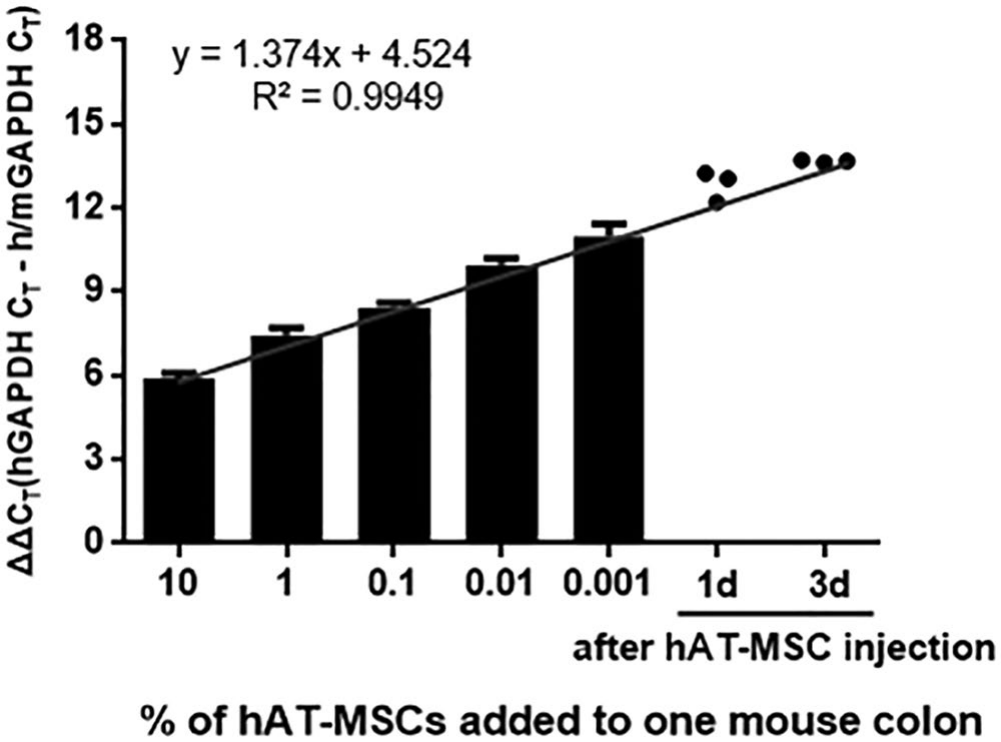
Intraperitoneally administered hAT-MSCs do not migrate into the colon. Serial dilutions of hAT-MSCs were administered to the investigated mice and the expression of human-specific GAPDH was evaluated. The results are representative of three independent experiments.

Next, we tracked and quantified the intraperitoneally administered hAT-MSCs (2 × 10^6^ cells) by constructing standard curves using quantitative reverse transcription-PCR (qRT-PCR) (Fig. 4, Supplementary Fig. S3, and Supplementary Table S1). One day after hAT-MSC administration, approximately 0.002%, 0.12%, 0.02%, 0.06%, and 0.09% of the cells were detected in the heart, lung, liver, spleen, and kidney of the colitis mice, respectively (Supplementary Fig. S4). Three days after hAT-MSC injection, these percentages were lower than they were at day 1 (Supplementary Fig. S4). However, intraperitoneally infused hAT-MSCs were hardly detected in the brain and the inflamed colon tissues of the colitis mice at both day 1 and day 3 (Fig. 4 and Supplementary Fig. S4).

### hAT-MSC-produced TSG-6 induces the phenotypic switch to M2 macrophages *in vitro*

**Figure 5:**
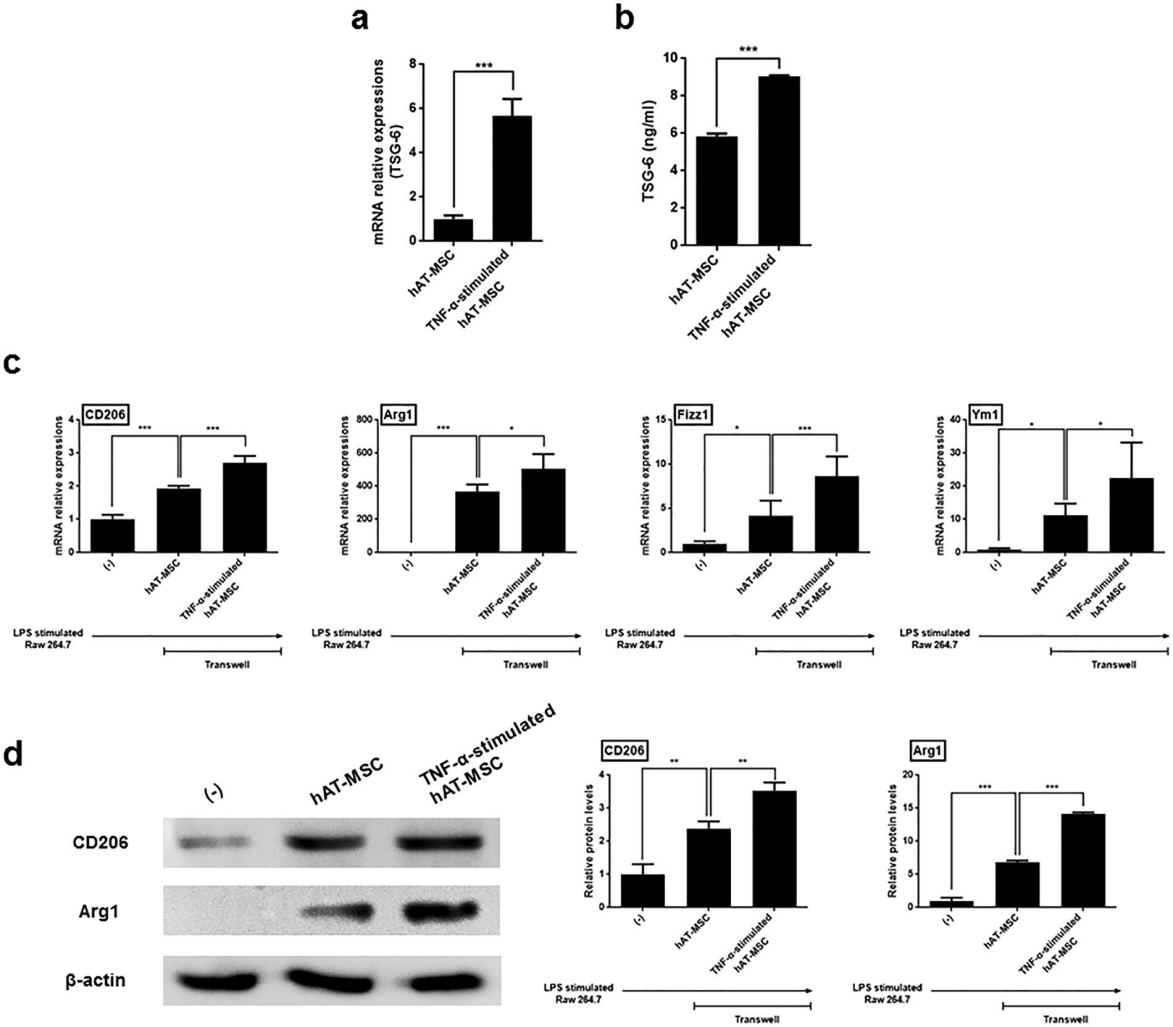
TNF-α-stimulated hAT-MSCs induce the expression of the M2 macrophage markers. (**a**,**b**) TSG-6 (**a**) gene and (**b**) protein expression levels in TNF-α-stimulated hAT-MSCs and naïve hAT-MSCs. (**c**,**d**) LPS-stimulated Raw 264.7 macrophages were co-cultured with naïve or TNF-α-stimulated hAT-MSCs in a transwell system for 48 h. (**c**) CD206, Arg1, Fizz1, and Ym1 mRNA expression levels. (**d**) CD206 and Arg1 protein expression levels in these cells. Results are presented as mean ± standard deviation of the data obtained in three independent experiments. *P < 0.05, ***P < 0.001.

We further investigated whether TSG-6, one of well-known immunomodulatory factors secreted by hAT-MSCs, could alter the macrophage phenotype to M2. To test the hypothesis, TNF-α-stimulated hAT-MSCs were used. TSG-6 gene expression was considerably induced in these cells, in comparison with that in the naïve hAT-MSCs (Fig. 5a). Additionally, TSG-6 concentration determined in the supernatant collected from the culture of TNF-α-stimulated hAT-MSCs was significantly higher than that measured using the naïve hAT-MSCs (Fig. 5b). Afterwards, lipopolysaccharide (LPS)-stimulated Raw 264.7 cells were co-cultured with the naïve or TNF-α-stimulated hAT-MSCs in a transwell system for 48 h, and M2 macrophage marker gene expression levels were shown to be significantly increased in the LPS-stimulated Raw 264.7 cells co-cultured with naïve hAT-MSCs, compared with those determined using the LPS-stimulated Raw 264.7 cells alone (Fig. 5c). Interestingly, the expression levels of the M2 macrophage markers were shown to be further increased in the LPS-stimulated Raw 264.7 cells co-cultured with TNF-α-stimulated hAT-MSCs, in comparison with those in the cells co-cultured with naïve hAT-MSCs (Fig. 5c). CD206 and Arg1 levels were determined as well, and LPS-stimulated Raw 264.7 cells co-cultured with naïve hAT-MSCs was shown to express CD206 and Arg1 (Fig. 5d). Furthermore, the expression of both proteins was significantly increased in LPS-stimulated Raw 264.7 cells co-cultured with TNF-α-stimulated hAT-MSCs compared with that in the cells co-cultured with naïve hAT-MSCs (Fig. 5d).

**Figure 6:**
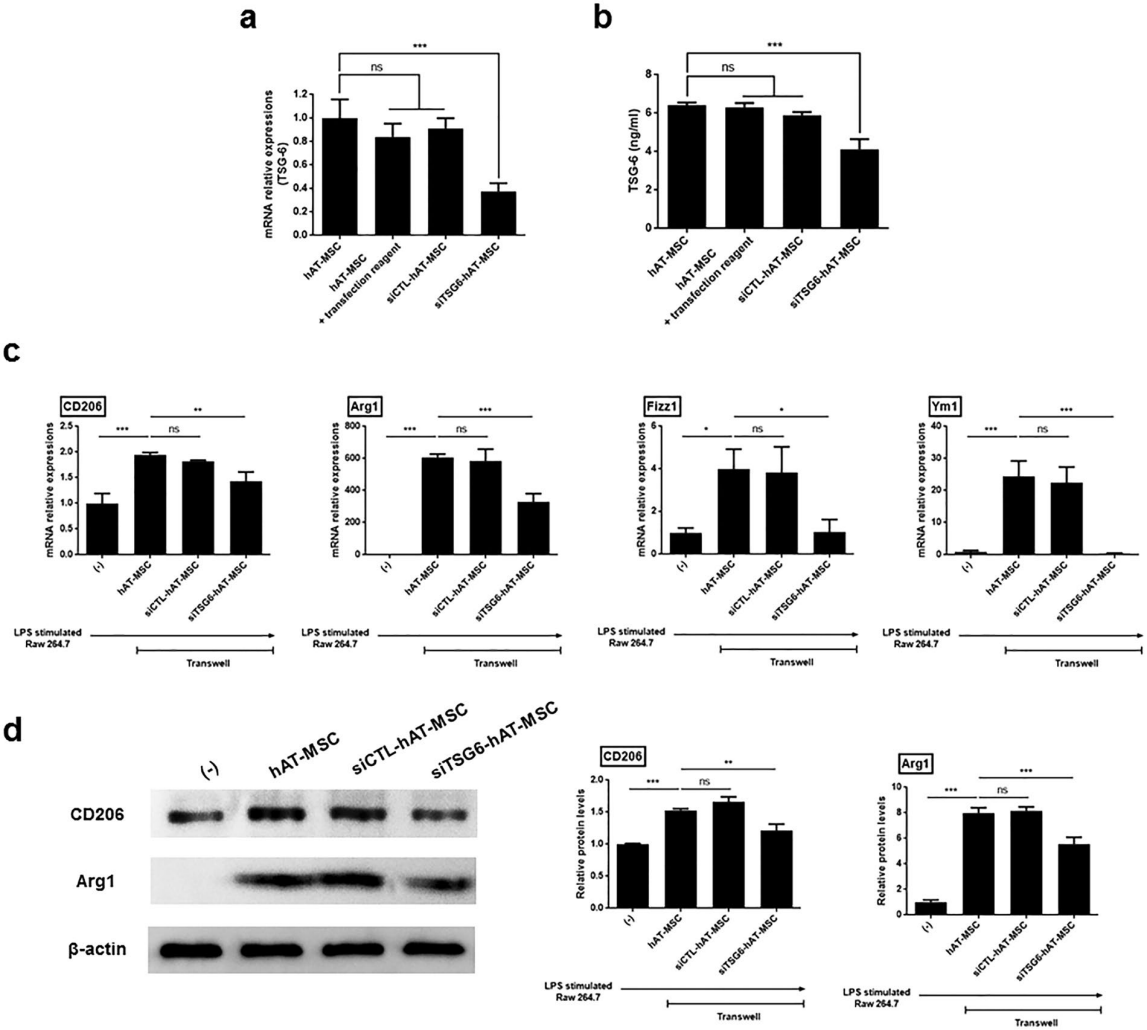
Expression of M2 macrophage markers decreases in hAT-MSCs transfected with TSG-6 siRNA. (**a**,**b**) TSG-6 (a) mRNA and (**b**) protein expression levels in hAT-MSCs transfected with TSG-6 siRNA (siTSG6-hAT-MSCs), hAT-MSCs transfected with control siRNA (siCTL-hAT-MSCs), hAT-MSCs with transfection reagent, and naïve hAT-MSCs. (**c**,**d**) LPS-stimulated Raw 264.7 macrophages were co-cultured with naïve or siCTL- or siTSG6-hAT-MSCs for 48 h. (c) CD206, Arg1, Fizz1, and Ym1 mRNA expression levels in these cells. (**d**) CD206 and Arg1 protein expression levels. Results are presented as mean ± standard deviation obtained in three independent experiments. *P < 0.05, **P < 0.01, ***P < 0.001.

Furthermore, TSG-6 expression in hAT-MSCs was knocked down after transient transfection with small interfering RNAs (siRNAs). hAT-MSCs transfected with siRNAs maintained their fibroblast-like shape, proliferative ability, and differentiation potential *in vitro* (Supplementary Fig. S2). TSG-6 expression was shown to be suppressed in hAT-MSCs transfected with TSG-6 siRNA (siTSG6-hAT-MSCs), whereas there was no significant change in TSG-6 expression levels in hAT-MSCs transfected with the control siRNA (siCTL-hAT-MSCs), compared with those in the naïve hAT-MSCs (Fig. 6a). Additionally, the concentration of TSG-6 protein secreted by siTSG6-hAT-MSCs was significantly decreased compared with that by the naïve or siCTL-hAT-MSCs (Fig. 6b). Afterwards, we co-cultured LPS-stimulated Raw 264.7 cells with naive or siRNA transfected hAT-MSCs in a transwell system for 48 h, and showed that M2 macrophage marker gene expression levels were significantly reduced in LPS-stimulated Raw 264.7 cells co-cultured with siTSG6-hAT-MSCs compared with those in the naïve hAT-MSCs (Fig. 6c). Furthermore, CD206 and Arg1 levels were significantly decreased as well in the LPS-stimulated Raw 264.7 cells co-cultured with siTSG6-hAT-MSCs (Fig. 6d). In contrast to this, siCTL-hAT-MSCs, with normal TSG-6 secretion levels, did not affect the gene or protein expression of M2 macrophage markers in LPS-stimulated Raw 264.7 cells (Fig. 6c and d).

### Intraperitoneal administration of hAT-MSCs with TSG-6 knockdown cannot alleviate IBD

**Figure 7:**
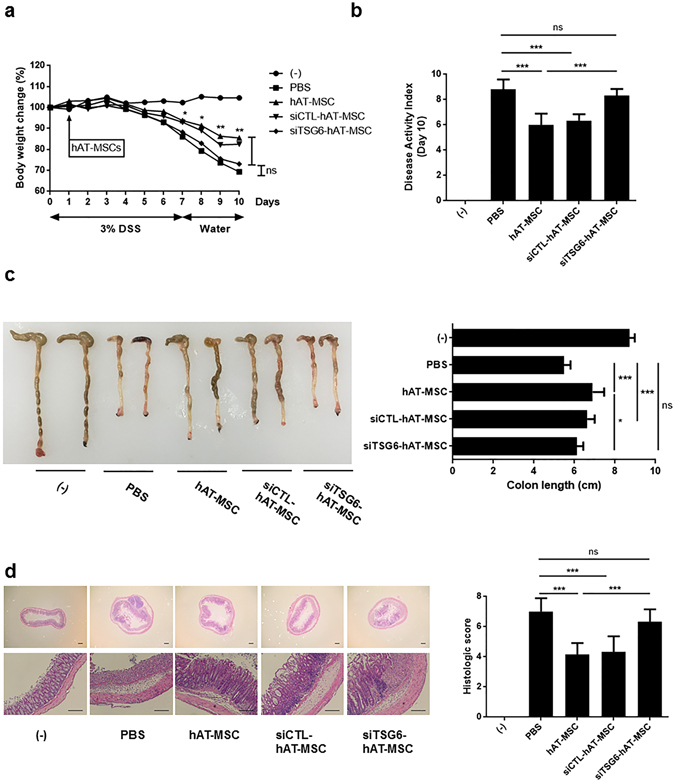
TSG-6 knockdown in hAT-MSCs inhibits their effect on IBD. (**a**) Body weight of animals injected with siTSG6-hAT-MSCs, siCTL-hAT-MSCs, or naïve hAT-MSCs was measured every day during the experiment, and expressed in terms of the relative change from the weight measured on day 0. (**b**–**d**) Mice were sacrificed on day 10 to evaluate clinical severity. (**b**) DAI, (**c**) colon length, and (**d**) representative colon tissue sections stained with H&E and histological scores are shown. Bars = 100 µm. Six to eight mice per group were used. Results are presented as mean ± standard deviation. *P < 0.05, **P < 0.01, ***P < 0.001.

We further investigated whether siRNA-transfected hAT-MSCs can alleviate DSS-induced colitis in mice. siTSG6-hAT-MSCs had no effect on body weight loss and DAI. In contrast, siCTL-hAT-MSCs were shown to significantly reduce body weight loss and DAI (Fig. 7a and b). Additionally, no significant improvement in the length and histologic score of colons obtained from the mice that received siTSG6-hAT-MSCs was observed. However, the administration of siCTL-hAT-MSCs led to a significant improvement in length and histologic score of these mice (Fig. 7c and d).

**Figure 8:**
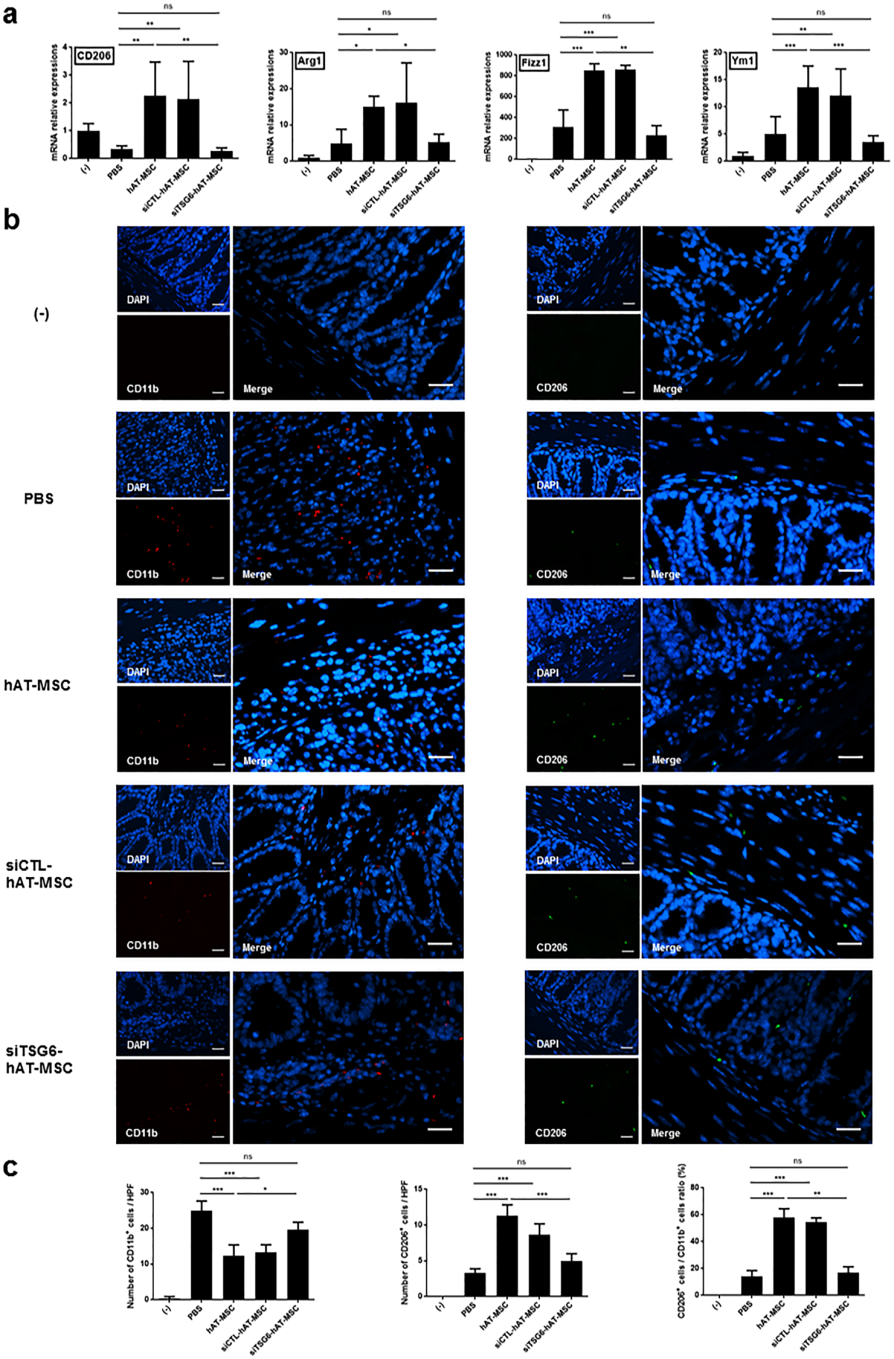
TSG-6 knockdown in hAT-MSCs inhibits their effect on M2 macrophage phenotypic switch. (**a**) The gene expression levels of CD206, Arg1, Fizz1, and Ym1 in the colon samples of mice injected with siTSG6-hAT-MSCs, siCTL-hAT-MSCs, or naïve hAT-MSCs. (**b**) Representative immunofluorescence staining using CD11b- or CD206-specific antibodies. Bars = 30 µm. (**c**) The number of CD11b- or CD206-positive cells within the inflammatory infiltrates, and the calculated percentage of CD206-positive cells among the CD11b-positive cells. Six to eight mice per group were used. Results are presented as mean ± standard deviation. *P < 0.05, **P < 0.01, ***P < 0.001.

Next, we assessed the expression level of M2 macrophages in the colons of colitis mice. Intraperitoneal administration of siTSG6-hAT-MSCs had no effect on the expression level of the M2 macrophage markers in the colon, compared with the levels measured in PBS-treated mice (Fig. 8a). In contrast, injection of siCTL-hAT-MSCs caused a significant increase in the expression of M2 macrophage markers in the colon (Fig. 8a). Furthermore, the percentage of CD206-positive M2 macrophages among the CD11b-positive total macrophages was significantly increased in the colon tissue sections of mice injected with siCTL-hAT-MSCs, whereas no significant difference was observed in the percentage of M2 macrophages in the colons of mice injected with siTSG6-hAT-MSCs compared with that in the PBS-treated mice (Fig. 8b and c).

## Discussion

Recently, hAT-MSCs, which can be obtained relatively easily in large quantities, have shown promising anti-inflammatory effects in studies that used different inflammatory disease models^25–29^. For chronic diseases, such as IBD, hAT-MSC administration may represent an important treatment option. Here, we showed that the application of hAT-MSCs to DSS-induced colitis mouse model may alleviate the symptoms of this disease, and that the weight loss and DAI were improved. Furthermore, by measuring the length of colon and assigning the scores based on the microscopic observation of the colon tissue obtained during the mouse autopsy, we showed that the intraperitoneal administration of hAT-MSCs has therapeutic effects against DSS-induced colitis in mice. In addition, we analysed the mechanisms underlying this process, in order to help the development of efficient cell therapies and their clinical application.

For these experiments, we injected human MSCs into an immunocompetent mouse model of IBD. We focused on the immune cells of mice after treatment with human MSCs. The human MSCs were also immunoprivileged, partly due to the low expression of major histocompatibility complex class II molecules^30^. Similar strategies involving the application of human MSCs to immunocompetent animal models have been adopted by several groups, and no obvious cross-species-induced immunological responses have been reported^15, 16, 31, 32^.

Macrophages observed in the inflamed tissues are either M1 or M2 macrophages, with unique roles in the regulation of inflammatory responses^33–37^. Macrophages have very important roles in innate and acquired immune response, and consequently, changes in macrophage type have a major impact on the inflamed tissue. M2 macrophages are known to be induced by IL-4 and IL-13, which also induce the expression of CD206, Arg1, Fizz1, and Ym1^21, 38^. Furthermore, recent studies have indicated that MSCs might have the ability to elicit a phenotypic switch from M1 to M2 macrophages *in vitro* as well as in animal models of acute kidney injury, spinal cord injury, and skin wound^22–24, 39–41^. Consistent with these reports, we found that colon tissues obtained from the hAT-MSC-injected group showed a considerably higher expression of M2 macrophage markers than those obtained from the PBS-treated group. The percentage of M2 macrophages among the total macrophages identified using immunofluorescence (IF) staining in the colon tissue sections was also higher in the hAT-MSC-treated group than in the PBS-treated group. The gene expression levels of pro-inflammatory cytokines, TNF-α, IL-1β, IFN-γ, and IL-17, were significantly reduced in the colons of the hAT-MSC-treated group, while that of anti-inflammatory cytokine IL-10 was significantly increased. These findings indicated that the intraperitoneal administration of hAT-MSCs could modulate the expression of inflammatory cytokines by inducing cell differentiation into M2 macrophages in the colon, thus alleviating the inflammatory responses. In addition, our results supported previous findings that MSCs infused into colitis mice reduced inflammation by altering macrophages^42, 43^.

Based on previous studies that revealed the therapeutic effects of MSCs in DSS-induced colitis mice, we determined the optimal procedure, that is, 2 × 10^6^ hAT-MSCs were administered intraperitoneally^32, 44^. We also examined the distribution of intraperitoneally injected hAT-MSCs using qRT-PCR. At 1 and 3 days after hAT-MSC administration, less than 0.5% of the injected cells were detected in the heart, lung, liver, spleen, kidney, and brain tissues. In addition, less than 0.001% of the injected cells were detected in colon tissues, despite inflammatory response. Similar results were obtained after administration of siCTL- and siTSG6-hAT-MSCs in colitis mice. These findings were consistent with those of several previous studies^17, 45^. Sala *et al*. had reported that less than 1% of the mouse bone marrow- and adipose-derived MSCs injected intraperitoneally into DSS-induced colitis mice were detected in the inflamed colon; however, a high frequency of injected cells formed aggregates with immune cells in the peritoneal cavity within 3 days^17^. Bazhanov *et al*. had also shown that the majority of human bone marrow-derived MSCs infused intraperitoneally into immunocompetent mice formed aggregates quickly and that these aggregates were attached to the peritoneal cavity^45^. Together with these results, our findings indicated that most of the intraperitoneally administered hAT-MSCs formed aggregates in the peritoneal cavity and reduced colitis by inducing M2 macrophages at sites distant from the inflamed colon through secretory factors.

Consistent with these findings, recent investigations have revealed that MSCs regulate the inflammatory processes through various soluble factors such as indoleamine 2,3-dioxygenase, TGF-β, prostaglandin E2 (PGE2), and TSG-6^31, 46–48^. Among these, TSG-6 has been shown to be pivotal for the anti-inflammatory effects of MSCs in corneal inflammation, severe burn injury, acute lung injury, acute peritonitis, pancreatitis, and IBD^13, 16, 17, 47, 49, 50^. In addition, Mittal *et al*. had recently shown that TSG-6 prevented lung injury by inducing a macrophage phenotype switch, although the TSG-6 used in this study was not released from stem cells^51^. Based on these previous findings and our results, it is tempting to speculate that TSG-6 secreted from hAT-MSCs plays a key role in inducing M2 macrophage polarization in the inflamed colon.

Therefore, we conducted *in vitro* experiments to examine whether the TSG-6 secreted by hAT-MSCs had the ability to induce macrophage phenotype switch. LPS-stimulated Raw 264.7 cells exhibit a conventional M1 macrophage pattern, and these cells were co-cultured with hAT-MSCs with TNF-α-induced or inhibited TSG-6 expression, or with the naive hAT-MSCs. The co-culturing of TSG-6 overexpressing hAT-MSCs with M1 macrophages led to a significant increase in the expression of M2 macrophage markers. However, because it is possible that other factors secreted by hAT-MSCs may affect this process as well, we performed additional experiments using TSG-6 targeting siRNAs. hAT-MSCs with TSG-6 knockdown were cultured with M1 macrophages, and the expression of M2 macrophage markers was shown to be decreased in these cells. Taken together, these findings suggest that TSG-6 secreted by hAT-MSCs plays a crucial role in the regulation of M1 to M2 phenotypic switch *in vitro*.

In order to analyse the effects of TSG-6 released by hAT-MSCs *in vivo*, we administered siRNA-treated hAT-MSCs to the experimental animals, and showed that, compared to the control group, the siTSG6-hAT-MSC-treated group showed no significant changes in the expression level of M2 macrophage markers in the colon. However, the expression of these markers was shown to be induced in the siCTL-hAT-MSC-treated group and naive hAT-MSC-treated group, in comparison with that in the PBS-treated group. Taken together, we confirmed that TSG-6 secreted by hAT-MSCs plays an important role in the alteration of macrophages to M2 phenotype in the inflamed mouse colons. Moreover, the change in the percentage of M2 macrophages among the total macrophages in the colon affected the severity of the DSS-induced colitis symptoms in mice. The siTSG6-hAT-MSC-treated group showed no improvement in weight loss rate, DAI, colon length, and histologic scores, compared to the PBS-treated group; however, the animals treated with siCTL-hAT-MSCs showed considerable improvement in these parameters, which achieved a level similar to those in the naive hAT-MSC-treated group.

Even so, we could not exclude the possibility that one or more other secretory factors released from hAT-MSCs could have increased ability to induce M2 macrophage polarization in colitis mice. Interestingly, recent investigations have shown that human MSCs could alter macrophage phenotype by producing PGE2 *in vitro* ^40, 52^. Additional experiments related to other MSC-secreted factors such as PGE2 are required to determine their effects on macrophages in IBD models. However, our findings suggested that TSG-6 plays a key role in M2 macrophage polarization *in vitro* and in DSS-induced colitis mice.

In conclusion, we presented a possible mechanism underlying the effects induced by TSG-6 in various inflammatory disease models, including IBD, which have been previously described. We demonstrated that hAT-MSC-secreted TSG-6 induced macrophages that infiltrated into the colon to switch to the M2 phenotype, thus regulating the expression of inflammatory cytokines and the alleviation of DSS-induced colitis symptoms in mice. These findings could help in the development of stem cell therapies for the treatment of IBD.

## Methods

### Cell preparations

Human adipose tissue was obtained from the abdominal subcutaneous fat of donor that provided an informed, written consent for research use. Cells isolated were prepared under a protocol approved by the Institutional Review Board of the R Bio (IRB No. RBIO-2015-04-002) as described in the Supplementary Methods section. Before their use in experiments, the expression of several stem cell markers on these cells was determined by flow cytometry (Supplementary Fig. S1). Additionally, cellular differentiation was observed (Supplementary Fig. S2), and the isolated hAT-MSCs at passage 3–5 were used in the following experiments.

RAW 264.7 cells, murine macrophage-like cell line, were purchased from the Korean Cell Line Bank (Seoul, Korea). Macrophages were cultured in Dulbecco’s Modified Eagle’s Medium (DMEM; PAN Biotech, Aidenbach, Germany) containing 10% foetal bovine serum (FBS; PAN Biotech) at 37 °C in a humidified atmosphere with 5% CO_2_.

### Stimulation of hAT-MSCs with TNF-α

Before further co-culture experiments, hAT-MSCs were stimulated with TNF-α, in order to induce TSG-6 expression. hAT-MSCs were plated at the density of 2 × 10^5^ cells per well in six-well plates or 1 × 10^6^ in 100-mm culture dishes, and incubated for 24 h. Afterwards, the medium was changed and 50 ng/mL of the recombinant human TNF-α (PeproTech, Rocky Hill, NJ, USA) was added, and the incubation was continued for another 24 h. The mRNA expression levels of TSG-6 in these cells were determined using qRT-PCR.

### Transfection of hAT-MSCs with siRNA

When they reach approximately 40% confluence, hAT-MSCs were transfected with TSG-6 siRNA or control siRNA (sc-39819 and sc-37007, respectively; Santa Cruz Biotechnology, Santa Cruz, CA, USA) for 48 h using Lipofectamine 2000 (Invitrogen, Carlsbad, CA, USA) according to the manufacturers’ instructions. TSG-6 knockdown was confirmed in these cells using qRT-PCR before they were used for further experiments.

### Animal experiments

Male C57BL/6 J mice aged 6-8 weeks were purchased from the Central Lab Animal Inc. (Seoul, Korea) and they were housed under controlled conditions of temperature (20 °C), humidity (50%) and light cycle (7 am lights on, 7 pm lights off). The study and all experimental procedures involving animals were approved by the Institutional Animal Care and Use Committee of Seoul National University (SNU-151007-3-1), and the animal study protocol was performed in accordance with the approved guidelines. Colitis was induced by the *ad libitum* administration of 3% DSS (36–50 kDa, MP biomedical, Solon, OH, USA) in the drinking water from day 0 to day 7. On day 1, the following procedure was performed: hAT-MSCs (2 × 10^6^ cells in 200 µL PBS) or the identical PBS volume were injected intraperitoneally into mice; 2 × 10^6^ hAT-MSCs transfected with TSG-6 siRNA in 200 µL of PBS, 2 × 10^6^ hAT-MSCs transfected with scrambled siRNA control in 200 µL of PBS, 2 × 10^6^ control hAT-MSCs in 200 µL of PBS, or the identical volume of PBS was injected intraperitoneally into mice for systemic siRNA-hAT-MSC experiments. hAT-MSCs transfected with si-RNA were used immediately after the transfection protocol described above was completed. In each experiment, mice receiving normal drinking water were used as the naïve group. Body weight of each mouse was measured every 24 h. The mice were sacrificed on day 10, and the colon samples were collected for further processing.

### Assessment of colitis severity

The DAI was calculated by scoring the body weight loss (grades, 0–4: 0, none; 1, < 10% loss of the initial body weight; 2, 10–15% loss of the initial body weight; 3, 15–20% loss of the initial body weight; 4, > 20% loss of the initial body weight), stool consistency (grades, 0–2: 0, none; 1, mild diarrhoea; 2, moderate to severe diarrhoea), rectal bleeding (grades, 0–2: 0, none; 1, mild bleeding; 2, moderate to severe bleeding), and general activity (grades, 0–2: 0, normal; 1, mildly depressed; 2, moderately to severely depressed).

### Histological analysis

Colon samples were fixed in 10% formaldehyde for 24 h, embedded in paraffin, and cut into 4-μm sections, which were stained with H&E. A total of 30 fields per group was selected randomly and histological examinations were performed in a blinded manner (magnification, 200 × ). The severity of symptoms was determined by scoring the extent of bowel wall thickening (grades, 0–3: 0, none; 1, mucosa; 2, mucosa and submucosa; 3, transmural), the damage of crypt (grades, 0–3: 0, none; 1, loss of goblet cells; 2, only surface epithelium intact; 3, loss of entire crypt and epithelium), and the infiltration of inflammatory cells (grades, 0–2: 0, none; 1, mild to moderate; 2, severe).

### Co-culturing of Raw 264.7 macrophages with hAT-MSCs

Raw 264.7 cells were stimulated with 200 ng/mL of LPS (Sigma-Aldrich, St. Louis, MO, USA) for 6 h before further experiments. The LPS-stimulated macrophages were plated at the density of 1 × 10^6^ cells per well in six-well plates and 2 × 10^5^ hAT-MSCs, control siRNA-hAT-MSCs, or TSG-6 siRNA-hAT-MSCs were seeded onto 0.4-μm pore-sized transwell inserts (SPL Life Science, Pocheon, Korea). After 48 h of incubation, total RNA and proteins were extracted from the Raw 264.7 macrophages following their trypsinization.

### RNA extraction, cDNA synthesis, and qRT-PCR

Total RNA was extracted from homogenised colon tissue or Raw 264.7 cells using the Easy-BLUE Total RNA Extraction kit (Intron Biotechnology, Seongnam, Korea) according to the manufacturer’s instructions. cDNA was synthesised using LaboPass M-MuLV Reverse Transcriptase (Cosmo Genetech, Seoul, Korea) and the samples were analysed in duplicate using 10 µL of AMPIGENE qPCR Green Mix Hi-ROX with SYBR Green dye (Enzo Life Sciences, Farmingdale, NY, USA) and 400 nM forward and reverse primers (Cosmo Genetech). Expression levels of the target genes were normalised to that of glyceraldehyde 3-phosphate dehydrogenase (GAPDH). Primer sequences used in this study are listed in Supplementary Table S2.

### Determination of TSG-6 expression by MSCs in the conditioned medium (CM)

TSG-6 secreted by naïve hAT-MSCs, TNF-α-stimulated hAT-MSCs, siCTL-hAT-MSCs, or siTSG6-hAT-MSCs grown in the CM was quantified by ELISA kit (MyBiosource, San Diego, CA, USA), according to the manufacturer’s instructions.

### Western blot analysis

Total proteins were extracted from Raw 264.7 cells using PRO-PREP Protein Extraction Solution (Intron Biotechnology) according to the manufacturer’s instructions. The concentrations of the protein samples were measured using Bio-Rad DC Protein Assay Kit (Bio-Rad, Hercules, CA, USA). The proteins were separated by sodium dodecyl sulfate-polyacrylamide gel electrophoresis and transferred to polyvinylidene difluoride membranes (Millipore, Billerica, MA, USA). The membranes were blocked by 5% non-fat dry milk in Tris-buffered saline containing 0.1% Tween 20 and incubated with primary antibodies against CD206 (1:1000; Abcam, Cambridge, MA, USA) and Arg1 (1:1000; Cell Signaling Technology, Beverly, MA, USA) at 4 °C overnight. The membranes were incubated with secondary antibodies at room temperature for 1 h. The immunoreactive bands were visualised using enhanced chemiluminescence (Advansta, Menlo Park, CA, USA) and normalised to β-actin levels (Santa Cruz Biotechnology).

### IF analyses

Paraffin-embedded 4-μm thick colon tissue sections were deparaffinised in xylene and rehydrated sequentially in 100%, 95%, 80%, and 70% ethanol solutions. After antigen retrieval using 10 mM citrate buffer (Sigma-Aldrich), the sections were blocked with a blocking buffer containing 5% bovine serum albumin and 0.3% Triton X-100 (both from Sigma-Aldrich) for 1 h. The slides were then incubated overnight at 4 °C with antibodies against phycoerythrin-conjugated CD11b (1:100; Abcam) and CD206 (1:200; Santa Cruz Biotechnology). After three washes, the sections incubated with CD206 antibody were incubated with fluorescein isothiocyante-conjugated secondary antibody (1:200; Santa Cruz Biotechnology) for 1 h at 20 °C in the dark. The colon sections stained with antibody against either CD11b or CD206 were washed three times and mounted in a Vectashield mounting medium containing 4′,6-diamidino-2-phenylindole (Vector Laboratories, Burlingame, CA, USA). The samples were observed using EVOS FL microscope (Life Technologies, Darmstadt, Germany). Immunoreactive cells were counted in 20 random fields per group and the ratio of CD206-/CD11b-positive cells was calculated in colon sections from the same mice.

### Generation of the GAPDH standard curve

Standard curves for the evaluation of the migratory ability of intraperitoneally injected cells were generated by administering serial dilutions of hAT-MSCs to mouse tissues, as described previously^14, 16^. Briefly, 2 × 10, 2 × 10^2^, 2 × 10^3^, 2 × 10^4^, or 2 × 10^5^ hAT-MSCs were added to the whole mouse organs prior to homogenization. After the total RNA was extracted from the samples (Easy-BLUE Total RNA Extraction kit; Intron Biotechnology), cDNA was synthesised (LaboPass M-MuLV Reverse Transcriptase; Cosmo Genetech) using 1 μg of RNA. qRT-PCR using human-specific GAPDH primers (forward primer, 5′-TGC TTT TAA CTC TGG TAA AGT GGA TA-3′; reverse primer, 5′-GTG GAA TCA TAT TGG AAC ATG TAA AC-3′) was performed in order to generate the standard curve. The curve was corrected by performing parallel qRT-PCR with primers used to amplify both human and mouse GAPDH (forward primer, 5′-CAG CGA CAC CCA CTC CTC CAC CTT-3′; reverse primer, 5′-CAT GAG GTC CAC CAC CCT GTT GCT-3′).

### Statistical analysis

Data are shown as mean ± standard deviation. Group means were compared by one-way analysis of variance (ANOVA) using the GraphPad Prism v.6.01 software (GraphPad Inc., La Jolla, CA, USA). P value of < 0.05 was considered statistically significant.

## Electronic supplementary material

Supplementary Information

## References

[1] Bouma G, Strober W (2003). The immunological and genetic basis of inflammatory bowel disease. Nature Reviews Immunology.

[2] van Beelen Granlund A (2013). Whole genome gene expression meta-analysis of inflammatory bowel disease colon mucosa demonstrates lack of major differences between Crohn’s disease and ulcerative colitis. PLoS One.

[3] Knights D, Lassen KG, Xavier RJ (2013). Advances in inflammatory bowel disease pathogenesis: linking host genetics and the microbiome. Gut.

[4] Manichanh C, Borruel N, Casellas F, Guarner F (2012). The gut microbiota in IBD. Nature Reviews Gastroenterology and Hepatology.

[5] Neurath MF (2014). Cytokines in inflammatory bowel disease. Nature Reviews Immunology.

[6] Gonzalez-Rey, E., Gonzalez, M. A., Rico, L., Buscher, D. & Delgado, M. Human adult stem cells derived from adipose tissue protect against experimental colitis and sepsis. *Gut* (2009). 10.1136/gut.2008.168534 19136511

[7] Jung, K. H. *et al*. Human bone marrow–derived clonal mesenchymal stem cells inhibit inflammation and reduce acute pancreatitis in rats. *Gastroenterology* **140**, 998–1008 e1004 (2011). 10.1053/j.gastro.2010.11.047 21130088

[8] Liu Y (2010). Therapeutic potential of human umbilical cord mesenchymal stem cells in the treatment of rheumatoid arthritis. Arthritis research & therapy.

[9] Prockop DJ, Oh JY (2012). Mesenchymal stem/stromal cells (MSCs): role as guardians of inflammation. Molecular Therapy.

[10] Lee JW, Fang X, Krasnodembskaya A, Howard JP, Matthay MA (2011). Concise review: Mesenchymal stem cells for acute lung injury: role of paracrine soluble factors. Stem Cells.

[11] Silini A, Parolini O, Huppertz B, Lang I (2013). Soluble factors of amnion-derived cells in treatment of inflammatory and fibrotic pathologies. Current stem cell research & therapy.

[12] Choi H, Lee RH, Bazhanov N, Oh JY, Prockop DJ (2011). Anti-inflammatory protein TSG-6 secreted by activated MSCs attenuates zymosan-induced mouse peritonitis by decreasing TLR2/NF-kappaB signaling in resident macrophages. Blood.

[13] Danchuk S (2011). Human multipotent stromal cells attenuate lipopolysaccharide-induced acute lung injury in mice via secretion of tumor necrosis factor-α-induced protein 6. Stem cell research & therapy.

[14] Lee RH (2009). Intravenous hMSCs improve myocardial infarction in mice because cells embolized in lung are activated to secrete the anti-inflammatory protein TSG-6. Cell Stem Cell.

[15] Qi Y (2014). TSG-6 released from intradermally injected mesenchymal stem cells accelerates wound healing and reduces tissue fibrosis in murine full-thickness skin wounds. J Invest Dermatol.

[16] Roddy GW (2011). Action at a distance: systemically administered adult stem/progenitor cells (MSCs) reduce inflammatory damage to the cornea without engraftment and primarily by secretion of TNF-alpha stimulated gene/protein 6. Stem Cells.

[17] Sala E (2015). Mesenchymal stem cells reduce colitis in mice via release of TSG6, independently of their localization to the intestine. Gastroenterology.

[18] Locati M, Mantovani A, Sica A (2012). Macrophage activation and polarization as an adaptive component of innate immunity. Advances in immunology.

[19] Chávez-Galán L, Olleros ML, Vesin D, Garcia I (2015). Much more than M1 and M2 macrophages, there are also CD169 + and TCR + macrophages. Frontiers in immunology.

[20] Martinez FO, Gordon S, Locati M, Mantovani A (2006). Transcriptional profiling of the human monocyte-to-macrophage differentiation and polarization: new molecules and patterns of gene expression. The Journal of Immunology.

[21] Murray PJ, Wynn TA (2011). Protective and pathogenic functions of macrophage subsets. Nat Rev Immunol.

[22] Cho DI (2014). Mesenchymal stem cells reciprocally regulate the M1/M2 balance in mouse bone marrow-derived macrophages. Exp Mol Med.

[23] Geng Y (2014). Mesenchymal stem cells ameliorate rhabdomyolysis-induced acute kidney injury via the activation of M2 macrophages. Stem cell research & therapy.

[24] Nakajima H (2012). Transplantation of mesenchymal stem cells promotes an alternative pathway of macrophage activation and functional recovery after spinal cord injury. J Neurotrauma.

[25] Choi EW, Lee M, Song JW, Shin IS, Kim SJ (2016). Mesenchymal stem cell transplantation can restore lupus disease-associated miRNA expression and Th1/Th2 ratios in a murine model of SLE. Scientific Reports.

[26] González MA, Gonzalez-Rey E, Rico L, Büscher D, Delgado M (2009). Adipose-derived mesenchymal stem cells alleviate experimental colitis by inhibiting inflammatory and autoimmune responses. Gastroenterology.

[27] Lopez‐Santalla M (2015). Human Adipose‐Derived Mesenchymal Stem Cells Modulate Experimental Autoimmune Arthritis by Modifying Early Adaptive T Cell Responses. Stem Cells.

[28] Shin, T.-H. *et al*. Human adipose tissue-derived mesenchymal stem cells alleviate atopic dermatitis via regulation of B lymphocyte maturation. *Oncotarget* (2016). 10.18632/oncotarget.13473 PMC5352174 27888809

[29] van den Broek LJ (2013). Differential response of human adipose tissue-derived mesenchymal stem cells, dermal fibroblasts, and keratinocytes to burn wound exudates: potential role of skin-specific chemokine CCL27. Tissue Engineering Part A.

[30] Le Blanc K, Tammik C, Rosendahl K, Zetterberg E, Ringdén O (2003). HLA expression and immunologic propertiesof differentiated and undifferentiated mesenchymal stem cells. Experimental hematology.

[31] Kim HS (2015). Human Umbilical Cord Blood Mesenchymal Stem Cell‐Derived PGE2 and TGF‐β1 Alleviate Atopic Dermatitis by Reducing Mast Cell Degranulation. stem cells.

[32] Kim, H. S. *et al*. Human umbilical cord blood mesenchymal stem cells reduce colitis in mice by activating NOD2 signaling to COX2. *Gastroenterology* **145**, 1392–1403 e1391–1398, doi:10.1053/j.gastro.2013.08.033 (2013). 10.1053/j.gastro.2013.08.033 23973922

[33] Jang S-E, Han MJ, Kim S-Y, Kim D-H (2014). Lactobacillus plantarum CLP-0611 ameliorates colitis in mice by polarizing M1 to M2-like macrophages. International immunopharmacology.

[34] Nishikawa K (2014). Interleukin-17 induces an atypical m2-like macrophage subpopulation that regulates intestinal inflammation. PloS one.

[35] Wang Y (2007). *Ex vivo* programmed macrophages ameliorate experimental chronic inflammatory renal disease. Kidney international.

[36] Wynn TA, Chawla A, Pollard JW (2013). Macrophage biology in development, homeostasis and disease. Nature.

[37] Zhu W (2014). Disequilibrium of M1 and M2 macrophages correlates with the development of experimental inflammatory bowel diseases. Immunol Invest.

[38] Sica A, Erreni M, Allavena P, Porta C (2015). Macrophage polarization in pathology. Cellular and Molecular Life Sciences.

[39] Vasandan AB (2016). Human Mesenchymal stem cells program macrophage plasticity by altering their metabolic status via a PGE2-dependent mechanism. Sci Rep.

[40] Ylöstalo JH, Bartosh TJ, Coble K, Prockop DJ (2012). Human mesenchymal stem/stromal cells cultured as spheroids are self‐activated to produce prostaglandin E2 that directs stimulated macrophages into an anti‐inflammatory phenotype. Stem cells.

[41] Zhang QZ (2010). Human gingiva-derived mesenchymal stem cells elicit polarization of m2 macrophages and enhance cutaneous wound healing. Stem Cells.

[42] Anderson, P. *et al*. Adipose-derived mesenchymal stromal cells induce immunomodulatory macrophages which protect from experimental colitis and sepsis. *Gut*, gutjnl-2012-302152 (2012). 10.1136/gutjnl-2012-302152 22637701

[43] Parekkadan B (2011). Bone marrow stromal cell transplants prevent experimental enterocolitis and require host CD11b + splenocytes. Gastroenterology.

[44] Wang, M. *et al*. Intraperitoneal injection (IP), Intravenous injection (IV) or anal injection (AI)? Best way for mesenchymal stem cells transplantation for colitis. *Scientific Reports* **6** (2016). 10.1038/srep30696 PMC4973258 27488951

[45] Bazhanov N (2016). Intraperitoneally infused human mesenchymal stem cells form aggregates with mouse immune cells and attach to peritoneal organs. Stem Cell Res Ther.

[46] Gonzalo-Gil E (2016). Human embryonic stem cell-derived mesenchymal stromal cells ameliorate collagen-induced arthritis by inducing host-derived indoleamine 2, 3 dioxygenase. Arthritis research & therapy.

[47] Liu, L. *et al*. TSG-6 secreted by human umbilical cord-MSCs attenuates severe burn-induced excessive inflammation via inhibiting activations of P38 and JNK signaling. *Scientific Reports* **6** (2016). 10.1038/srep30121 PMC4957124 27444207

[48] Liu W, Zhang S, Gu S, Sang L, Dai C (2015). Mesenchymal stem cells recruit macrophages to alleviate experimental colitis through TGFβ1. Cellular Physiology and Biochemistry.

[49] He Z (2016). Intravenous hMSCs Ameliorate Acute Pancreatitis in Mice via Secretion of Tumor Necrosis Factor-α Stimulated Gene/Protein 6. Scientific Reports.

[50] Wang N (2012). Mesenchymal stem cells attenuate peritoneal injury through secretion of TSG-6. PLoS One.

[51] Mittal M (2016). TNFα-stimulated gene-6 (TSG6) activates macrophage phenotype transition to prevent inflammatory lung injury. Proceedings of the National Academy of Sciences.

[52] Vasandan AB (2016). Human Mesenchymal stem cells program macrophage plasticity by altering their metabolic status via a PGE2-dependent mechanism. Scientific Reports.

