# Supplementary material for: TSG-6 Secreted by Human Adipose Tissue-derived Mesenchymal Stem Cells Ameliorates DSS-induced colitis by Inducing M2 Macrophage Polarization in Mice
Source: Sci Rep. 2017 Jul 12;7:5187. doi: 10.1038/s41598-017-04766-7 (PMC5507867; doi:10.1038/s41598-017-04766-7)
Supplement: Supplementary file 1 — Supplementary Information [file 41598_2017_4766_MOESM1_ESM.pdf]

Supplementary Data for Song *et al*.

TSG-6 Secreted by Human Adipose Tissue-derived Mesenchymal Stem Cells Ameliorates Inflammatory Bowel Disease by Inducing M2 Macrophage Polarization in Mice

Woo-Jin Song[1], Qiang Li[1], Min-Ok Ryu[1], Jin-Ok Ahn[1], Dong Ha Bhang[2], Yun Chan Jung[3], Sung-Keun Kang[4], Hwa-Young Youn[1,*]

[1]Department of Veterinary Internal Medicine, College of Veterinary Medicine, Seoul National University, Seoul 08826, Republic of Korea

[2]Department of Molecular and Cellular Biology, Samsung Biomedical Research Institute, Sungkyunkwan University School of Medicine, Suwon, Gyeonggi 16419, Republic of Korea

[3]KPC Corporation, Gwangju, Gyeonggi 12773, Republic of Korea

[4]Biostar Stem Cell Research Institute, R Bio Co. Ltd., Seoul 08506, Republic of Korea

[*]Corresponding author: Hwa-Young Youn (*Tel:* +82-2-880-1266, *E-mail:* hyyoun@snu.ac.kr)

Supplementary Methods

hAT-MSCs isolation and culturing

hAT-MSCs were isolated and cultured under a protocol approved by the Institutional Review Board of the R Bio (IRB No. RBIO-2015-04-002). Subcutaneous adipose tissues were digested with collagenase I (1 mg/mL; Gibco/Life Technologies, Grand Island, NY, USA) under gentle agitation for 60 min at 37°C and filtered through a 100-mm nylon sieve to remove cellular debris, followed by centrifugation at 470 g for 5 min. After centrifugation, the pellet was resuspended in Dulbecco's modified eagle's medium (DMEM; Invitrogen, Carlsbad, CA, USA)–based media and cultured overnight at 37°C in a humidified atmosphere with 5% CO_2. After 24 h, the cell adhesion was checked under an inverted microscope, and non-adherent cells were removed by washing with phosphate buffered saline (PBS; PAN biotech, Aidenbach, Germany). The cell medium was changed to keratinocyte-serum free medium (SFM; Invitrogen) based media containing 0.2 mM ascorbic acid, 0.09 mM calcium, 5 ng/mL recombinant epidermal growth factor (rEGF; Prospec, East Brunswick, NJ, USA), and 5% fetal bovine serum (FBS; PAN biotech). The cells were maintained for 4 to 5 days until confluent (passage 0). When the cells reached 90% confluency, they were subculture-expanded in Keratinocyte-SFM-based media containing 0.2 mM ascorbic acid, 0.09 mM calcium, 5 ng/mL rEGF, and 5% FBS. Isolated hAT-MSCs were used at passage 3-5 for the following experiments.

Characterization of hAT-MSCs

Cells isolated were characterized for the expression of stem cell markers by flow cytometry using fluorescein isothiocyante (FITC)-, or phycoerythrin (PE)-conjugated antibodies against the following proteins: CD31-FITC, CD34-PE, CD45-FITC, CD73-PE, and CD90-PE (all

from BD Biosciences, Franklin Lakes, NJ, USA). Cells were analyzed using a FACSCalibur flow cytometer (BD Biosciences) with the CELLQuest software (BD Biosciences). Cellular differentiation was evaluated using the StemPro Adipogenesis Differentiation, StemPro Osteogenesis Differentiation, and StemPro Chondrogenesis Differentiation kits (all from Gibco/Life Technologies, Carlsbad, CA, USA) according to the manufacturer’s instructions followed by Oil Red O staining, Alizarin Red staining, and Alcian Blue staining, respectively.

Supplementary Figures

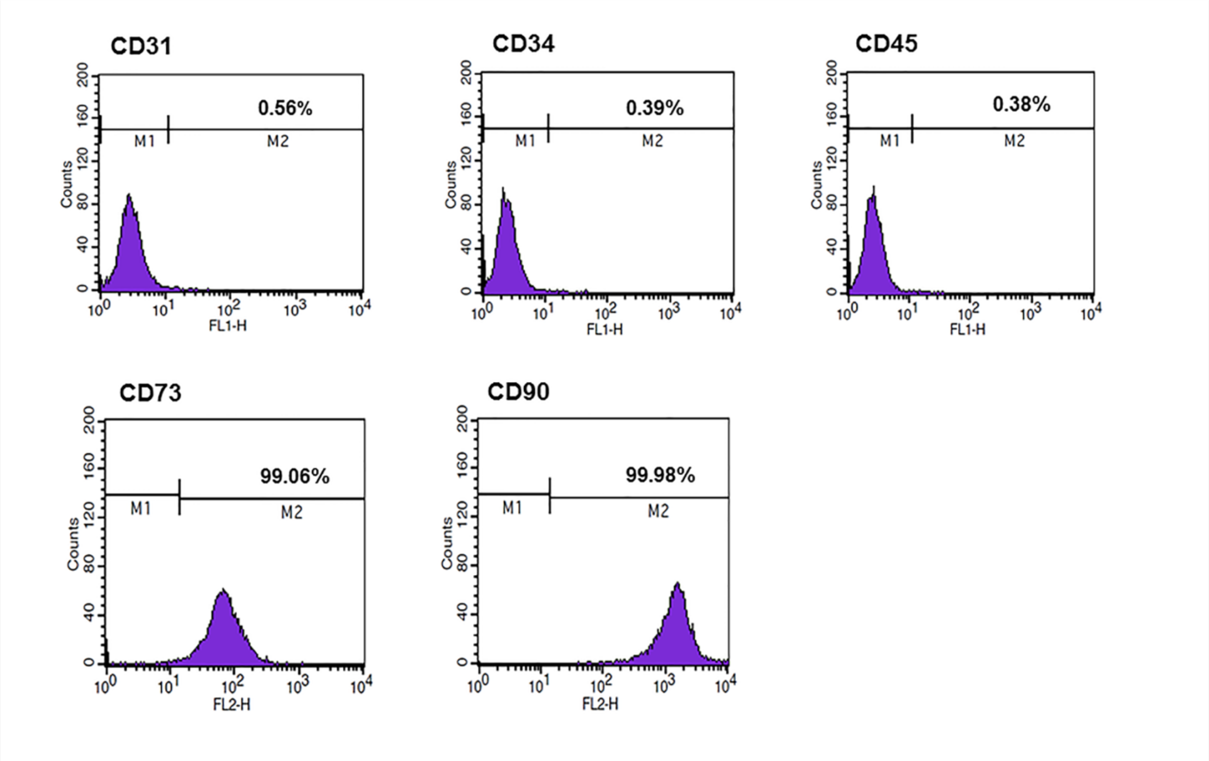

Figure S1. Immunophenotypic analysis by flow cytometry.

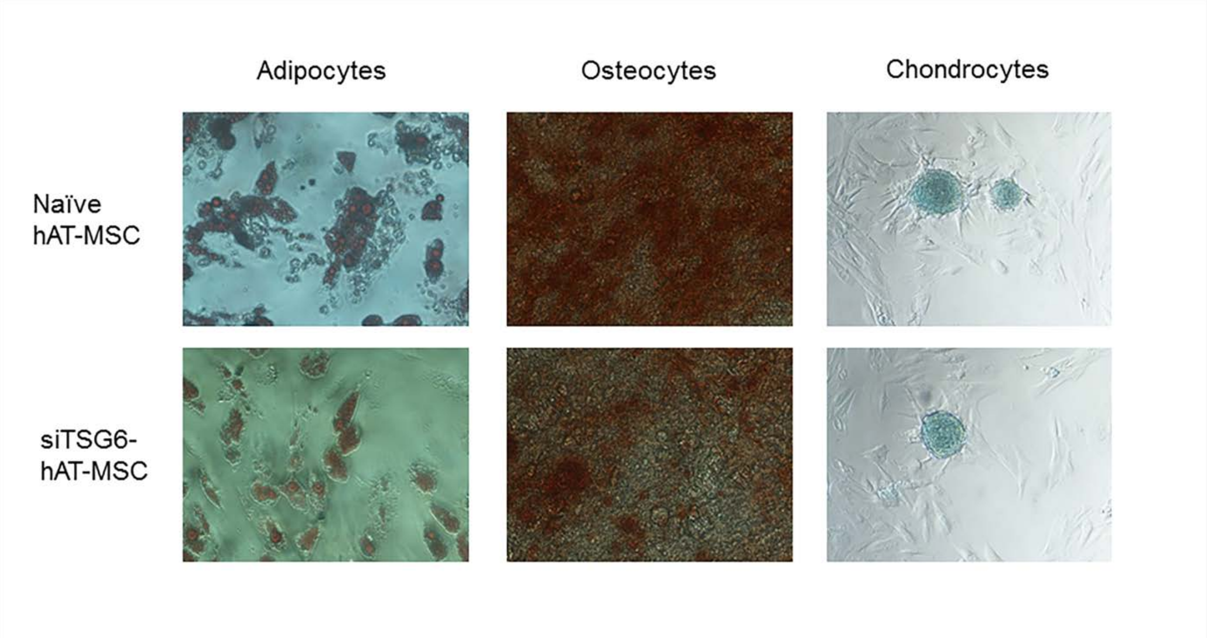

Figure S2. Adipogenic (Oil Red O staining), osteogenic (Alizarin Red S staining), and chondrogenic (Alcian Blue staining) differentiation of naïve and siTSG6-hAT-MSCs. Original magnification: 200×.

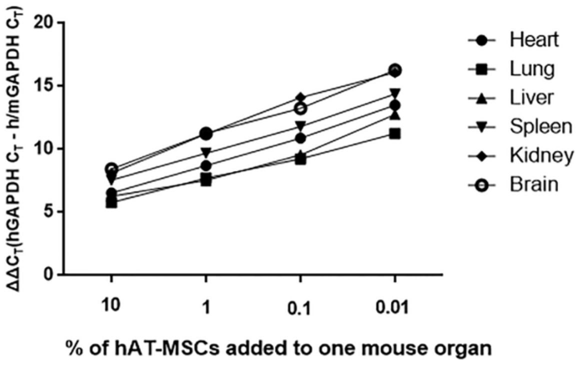

**Heart**	y = 2.3074x + 4.1409	(R^2 = 0.9979)
**Lung**	y = 1.7951x + 4.008	(R^2 = 0.9973)
**Liver**	y = 2.1481x + 3.6681	(R^2 = 0.9959)
**Spleen**	y = 2.2642x + 5.2034	(R^2 = 0.9975)
**Kidney**	y = 2.6842x + 5.678	(R^2 = 0.9907)
**Brain**	y = 2.559x + 5.915	(R^2 = 0.9945)

Figure S3. Standard curves for qRT-PCR assays of human mRNA for GAPDH.

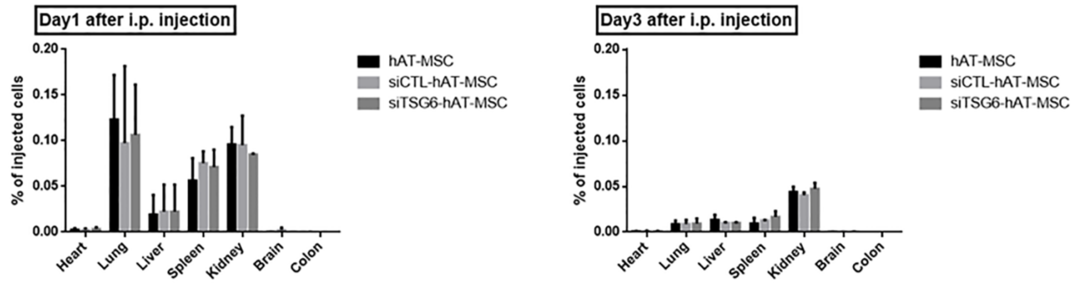

Figure S4. Distribution of hAT-MSCs in colitis mice after intraperitoneally infusion.

Supplementary Tables

Table S1. C_T values for human-specific and human/mouse GAPDH assessed by qRT-PCR in colons of mice

Number of hAT-MSCs added per one mouse colon	Animal number	C_T values of human-specific GAPDH	C_T values of human/mouse GAPDH	C_T of human-specific GAPDH – C_T of human/mouse GAPDH		
				Values	Average	Standard deviation
2×10^5	1	30.64795685	24.49607086	6.151885986	5.902929	0.174534
	2	30.58596039	24.50877762	6.07718277		
	3	30.34713745	24.51195145	5.797086716		
	4	30.4168148	24.61972809	5.835186005		
2×10^4	1	31.09445953	23.68227768	7.412181854	7.453283	0.246507
	2	30.93130112	23.7868824	7.144418716		
	3	32.85596657	25.4803009	7.375665665		
	4	33.04473114	25.51711082	7.527620316		
2×10^3	1	33.76989174	25.38811874	8.381772995	8.430801	0.167655
	2	33.84774399	25.52143478	8.326309204		
	3	33.9227066	25.33699036	8.585716248		
	4	33.9914608	25.54742622	8.444034576		
2×10^2	1	35.87288094	25.7082901	10.16459084	9.977036	0.231780
	2	35.60738182	25.7031498	9.904232025		
	3	35.2568512	25.41586876	9.840982437		
	4	35.40197563	25.43928909	9.962686539		
2×10^1	1	36.67917442	25.47019768	11.20897675	10.98940	0.451630
	2	37.36874008	26.19055176	11.17818832		
	3	36.39127731	25.70456123	10.68671608		
	4	36.91268539	26.04187775	10.87080765		
Mouse colon without hAT-MSCs	1	Not determined	23.12542534	-	-	-
	2	Not determined	23.19706535	-	-	-
	3	Not determined	22.9878273	-	-	-
	4	Not determined	23.22959709	-	-	-

Table S2. List for primers for qRT-PCR

Gene	Forward (5′-3′)	Reverse (5′-3′)
**<u>Mouse</u>**		
TNF-α	CCCTCACACTCAGATCATCTTCT	GCTACGACGTGGGCTACAG
IL-1β	GTCTTTCCCGTGGACCTTC	TGTTCATCTCGGAGCCTGT
IFN-γ	CTCTTCTTGGATATCTGGAGGAACT	GCTGTTGCTGAAGAAGGTAGTAATC
IL-17	GGTCAACCTCAAAGTCTTTAACTCC	GAGGGATATCTATCAGGGTCTTCAT
IL-10	GTGATTTTAATAAGCTCCAAGACCA	GATCATCATGTATGCTTCTATGCAG
CD206	AACGGAATGATTGTGTAGTTCTAGC	TACAGGATCAATAATTTTTGGCATT
Arg1	CAGAAGAATGGAAGAGTCAG	CAGATATGCAGGGAGTCACC
Fizz1	GAATCTATTGTGGAGAAAAAGGTCA	AGCCGTGATACTAGTACAGGAGAAA
Ym1	GTGTACTCACCTGATCTATGCCTTT	CAGGAGAGTTTTTAGCTCAGTGTTC
GAPDH	AGTATGTCGTGGAGTCTACTGGTGT	AGTGAGTTGTCATATTTCTCGTGGT
**<u>Human</u>**		
TSG-6	AAAAACTGGCATTATTGATTATGGA	CAGTAGCAGATTTGGTTATCTTCGT
GAPDH	TGCTTTTAACTCTGGTAAAGTGGATA	GTGGAATCATATTGGAACATGTAAAC
